# Behaviour-Based Husbandry—A Holistic Approach to the Management of Abnormal Repetitive Behaviors

**DOI:** 10.3390/ani8070103

**Published:** 2018-06-27

**Authors:** Heather Bacon

**Affiliations:** Jeanne Marchig International Centre for Animal Welfare Education, The Royal (Dick) School of Veterinary Studies, The University of Edinburgh, Easter Bush Veterinary Centre, Roslin, Midlothian EH25 9RG, UK; Heather.Bacon@ed.ac.uk

**Keywords:** zoo, behavior, welfare, stereotypy, abnormal repetitive behavior, enrichment

## Abstract

**Simple Summary:**

This paper outlines some of the barriers to implementing improved zoo animal welfare in practice, and proposes a new strategy for the development of behavioral husbandry routines focused on the management and mitigation of abnormal repetitive behaviors. Focusing on enhancing zoo animal welfare by integrating aspects of ecology, ethology and clinical animal behavior into a practical and comprehensive approach to behavior-based husbandry.

**Abstract:**

The field of zoo animal welfare science has developed significantly over recent years. However despite this progress in terms of scientific research, globally, zoo animals still face many welfare challenges. Recently, animal welfare frameworks such as the five domains or five needs have been developed and suggested to improve the welfare of zoo animals, but without practical guidance, such tools may remain abstract from the daily experience of zoo animals. Similarly specific practical strategies such as those for enrichment development exist, but their lack of holistic integration with other aspects of animal husbandry and behavioral medicine means that overall, good zoo animal welfare may still be lacking. This paper outlines some of the barriers to implementing improved zoo animal welfare in practice, and proposes a new strategy for the development of behavioral husbandry routines focused on the management and mitigation of abnormal repetitive behaviors. Focusing on enhancing zoo animal welfare by integrating aspects of ecology, ethology and clinical animal behavior into a practical and comprehensive approach to behavior-based husbandry.

## 1. Introduction

The issue of zoo animal welfare has received increasing attention over recent years [1,2,3]. Operant conditioning, enclosure design and environmental enrichment strategies have all been suggested to improve the welfare of zoo animals by reducing stereotypical behavior and alleviating stress. Such strategies have even been suggested to also improve the captive breeding and reintroduction success of wildlife species [4,5,6,7,8]. Thus, the use of these strategies has important consequences for both the animal welfare and conservation activities of zoological collections. Despite the recognition and wide-scale implementation of such strategies, however, concerns around global zoo animal welfare remain, and behavioral pathologies are common in many species [9,10,11]. This article outlines some of the barriers to delivering positive welfare experiences through holistic behavioral management strategies to zoo animals, and makes recommendations for institutional approaches towards improving zoo animal welfare using the example of Abnormal Repetitive Behaviors (ARBs), through targeted behavioral management.

## 2. Zoo Animal Welfare

### 2.1. Zoo Animal Welfare: A Global Perspective

There are estimated to be 10,000–12,000 zoos and animal parks in the world [12]. Less than 2.3% are estimated to be accredited [13]. The animal welfare committee of the World Association of Zoos and Aquaria (WAZA) estimates that up to 95% of the global zoo community may not be meeting good practice standards (WAZA animal welfare committee, pers. comm, October 2015) such as those outlined in Caring for wildlife, the WAZA animal welfare strategy [1]. The field of zoo animal welfare science has developed significantly over recent years [1,2,14], however these developments often seem to be region-specific and it is possible that as zoos in the Western world embrace technology and science in the assessment and improvement of animal welfare, we may further increase the distance between the activities and standards of the global zoo community as many countries struggle to provide good zoo animal welfare [15]. It may also be suggested that because of the varying standards of zoos around the world, the concept of a ‘Global zoo community’ is a challenging one, but in terms of assessing ‘standards’, the distinction is not so simple as accredited versus non-accredited zoos. There are significant differences in animal acquisition, animal husbandry practices, record-keeping and collection-planning activities even between regional zoo membership and accreditation schemes (European Association of Zoos and Aquaria joint Taxon Advisory Group Chairs meeting pers. comm., May 2018), and so it appears increasingly important that the various regional zoological associations work together as a global community to support and raise standards of practice.

Whilst standards should never be tailored to the lowest common denominator, it is essential that the global zoo community is able to speak a ‘common language’ in terms of the application of animal welfare science and clinical animal behavior to zoo animal husbandry strategies, combining both an evidence-based approach and a flexible and pragmatic application of husbandry strategies. To achieve this, it is necessary that staff working within zoos are both empathetic and knowledgeable about the species that they manage.

A large proportion of the global zoo community struggles to adequately meet the behavioral and welfare needs of the animals housed within their zoological collections (WAZA animal welfare committee, pers. comm, October 2015). Such zoos are more likely to be located in countries where resources to provide for zoo animal welfare are restricted, and where technological and research advances in the fields of zoo animal welfare science and enrichment are limited. Whilst specific capacity-building of zoo professionals can be impactful [15,16], there are also cultural, resource-based and knowledge limitations: Knowledge of the diverse and complex needs of the species housed in zoos may contribute one barrier to the ability of zoos to provide good animal welfare [17]. Developments in animal welfare science have historically been biased towards the agricultural industry [18,19], whilst animal welfare in zoos has relied upon taking remedial action, once indicators of poor welfare are identified in zoo animals [17]. Additionally, robust welfare indicators in zoo species have not been developed for many species [20]. Globally, zoos have not always had access to necessary animal husbandry information [21] and the English language literature bias may hinder dissemination of information internationally [18]. Universal frameworks for providing good animal welfare to zoo animals have been proposed [1,14], and whilst these often provide aspirational policies, they may not necessarily meet the needs of zoos in terms of accessibility of information or on-the-ground practicalities. Even in well-resourced countries such as the UK an education in animal behavioral science does not usually form part of the vocational or formal training of zookeepers [22].

### 2.2. Zookeepers and the Importance of Husbandry

The importance of the keeper–animal relationship in supporting good animal welfare in zoos cannot be understated. Animals in zoos can and do distinguish familiar from unfamiliar people and positive interactions with familiar people such as keepers can modify their responses to unfamiliar people [23]. Thus, interactions with keepers may influence how an animal is able to cope with other people more generally. Stockmanship is a term used to describe the management of animals and a good stockperson is someone who manages animals in a safe, effective and low-stress manner for both the stock-keeper and animals involved [24]. Two key components of zookeeper stockmanship have been described: (1) the attitude of keepers towards the animals, and (2) their knowledge and experience of the animals under their care [24]. Thus, the role of zookeepers is paramount, not just in assessing animal welfare, preventing and recognising abnormal repetitive behaviours (ARBs), developing effective enrichment strategies and administering medication, but also in facilitating an animal’s ability to cope with other stressors. Whilst research in zoos has formed a vital component of improving zoo animal welfare [25], as outlined earlier in this article, there are still gaps in terms of delivering practical animal care and zookeeper knowledge.

Zoo animals are reliant upon their keepers for access to the vast majority of their resources. As humans we make decisions on a daily basis about the access of zoo animals to food, sleeping quarters, off-show areas, enrichment and conspecifics as well as breeding opportunities, enclosure design, animal transfers, and healthcare. It has been suggested that the lack of agency and control that zoo animals have over their daily routines and resources may be a factor in the development of ARBs, and that offering animals greater choice and control over their space and resources may mitigate ARBs [10,26,27].

**Figure 1:**
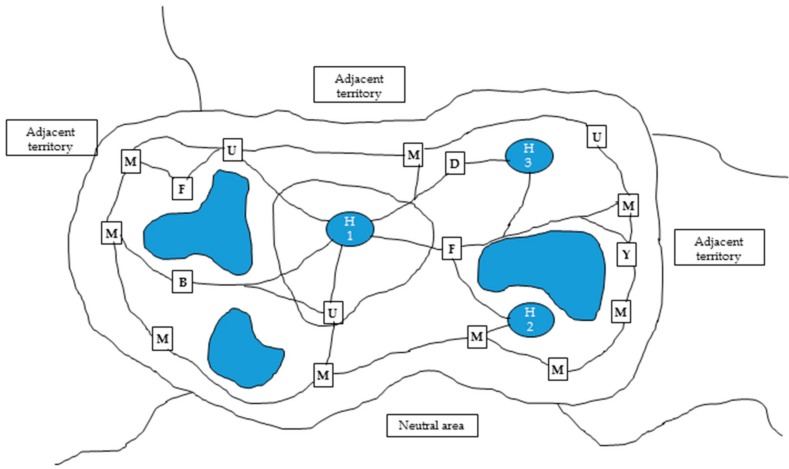
Schematic representation of an animal’s territory, adapted from [28,29] outlining that territories comprise specific resource-locations and travel paths between locations. H1: Primary refuge, H2: secondary refuge, H3: emergency concealment, B: bathing place, F: feeding place, U: urination/defecation place, M: demarcation place, D: drinking place, Y: food storage.

However, simply offering random choices may not be enough to mitigate ARBs as the choices and resources offered should make evolutionary sense to that particular animal and accommodate its species-specific behavioural needs. An animal’s territory may be defined as an area where an animal performs a specific set of acts (feeding, scent-marking, denning, resting, etc.) within specific locations [28,29] (Figure 1). Thus, it is important that the animal is housed in an environment that accommodates all of the resources and locations it needs to perform its normal behaviours at the appropriate times, and that a variety of resources are provided for animals that require multiple resources, e.g., denning sites in cheetahs [10]. Terrestrial animals display a normal level of ritualistic behaviour in their daily activities [28], and ARBs generally originate from frustration of these normal ritualistic motor patterns. Understanding the animal’s daily motor patterns and providing choice and control over resources that are important to it may help to mitigate the development and progression of ARBs by reducing behavioural frustration. Enclosures which are poorly designed and do not accommodate an animal with the space and choices of resources that it needs may not be environments in which that animal can cope, and thus it is essential that enclosures are designed to accommodate the animal’s natural ecology and behaviours, and that husbandry routines consider the animal’s biological rhythms, daily and seasonal routines.

Another important element of behavioral management is the maintenance of important social behaviors and appropriate social groupings. In a way similar to the categorization of enrichment items, humans tend to categorize animal species in terms of their social behavior—primarily in the binary categories of ‘solitary/unsocial’ or ‘social’ [30] and then in further social grouping, e.g., pairs, harems, etc. within this broader ‘social’ category. However, social behavior is better considered along a continuum of sociability rather than as a binary social versus unsocial [30].

For example, bear species have long been considered to be solitary in the wild despite the fact that they are well known to exchange information via scent [31,32], exhibit affiliative behaviors [33], share overlapping home-ranges [34] and engage in social interaction when resources are plentiful [33,35,36]. Even when competition for resources occurs in the wild, it is reported that non-aggressive interactions between bears are more common than aggressive interactions [37], indicating a level of social tolerance even between bear species. Social interactions in bears have also been reported in the zoo environment as a factor that reduces the risk of ARBs [38] and play, a suggested positive welfare indicator has been recorded in captive bears [39], these studies and others [40] suggest that social interaction may be beneficial as long as bears have an element of choice over social interactions and resources are plentiful [36]. Assessing a species’ sociability can be challenging, especially in cryptic species where the literature is deficient, but is worth intermittently critically evaluating the ecological literature, especially in species that do not thrive within current zoological management systems. For example, the fossa is generally described and housed as a solitary species in zoos, and yet reports of wild behavior include cooperative hunting, a behavior only described in gregarious species, and that “males are frequently observed in close and stable associations” [41]. In addition, this species displays complex polyandrous mating rituals {Lührs, 2010 #577}. Even wild giant pandas, a species long held in solitary conditions within zoos, demonstrate space-use in the wild indicative of a social species [42]. These finding have potential significance for progressing the development of social management of these species and the design of enclosures and husbandry routines to facilitate more social choice. In the case of Polar bears, significant developments in social grouping and enclosure design in the UK and Canada are reported to have resulted in improved behavioral health [43,44]. Considering this evidence, I suggest that zoos critically evaluate traditional husbandry routines with more dynamic and creative solutions aimed at providing for more extensive environmental and social choice, which may benefit both animal welfare and ex situ conservation success.

### 2.3. Back to Basics—The Importance of Behaviour in Zoo Animal Welfare

Zoo animal welfare is often linked to the behavior of zoo animals—and quite rightly so. The diversity and duration of behaviors demonstrated by a particular animal are useful in indicating its psychological state [45,46] and may also have implications for conservation success [47]. Of course, behavior alone does not always give us a comprehensive insight into an animal’s welfare state—for example, an animal may be behaving normally but may be physically unhealthy and thus its welfare may be compromised, but despite this potential limitation, an animal’s behavior often gives us important information about how that animal feels. Whilst traditionally zoos have focused on prioritizing the health of their animals, there is a recognition that health is not synonymous with welfare and that what matters to the animal is how it feels [48].

**Figure 2:**
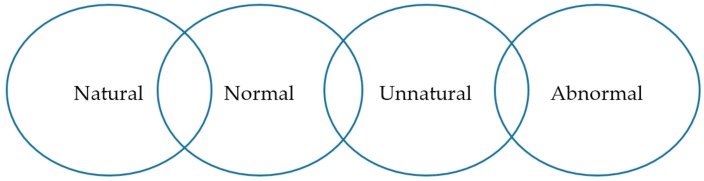
Schematic diagram of the overlaps of the four categories of behavior.

Animal behaviors may be classed into four overlapping categories: Natural behavior is typically observed in the wild, e.g., mating, foraging; Unnatural behavior may be normal or abnormal but is not typically observed in the wild, e.g., trained or learned behaviors; Normal behavior may be natural or unnatural but makes sense within the context in which it is performed, e.g., trained behaviors, interaction with enrichment items; Abnormal behavior is not typically seen in the wild and does not appear to serve a particular function, e.g., stereotypical behaviors [49] (Figure 2).

Understanding animal behavior is essential to the early detection of zoo animal welfare problems. It is recognized that a variety of abnormal repetitive behaviors (ARBs) comprising but not limited to stereotypic ritualistic behavior (pacing, head-tossing), self-directed behaviors (feather-plucking, over-grooming), or externally directed behaviors (conspecific mutilation, barrel-pouncing in polar bears), are common in many species held in zoos, e.g., canids, bears, elephants, primates, ruminants [9], birds [50,51] and even occur in less charismatic species such as fish and reptiles [46,52,53,54]. Often these ARBs may be dismissed merely as ‘behavior problems’ when in actuality their origin from repeated attempts to cope, behavioral frustration, or psychopathology [55] may actually indicate psychological distress and thus poor welfare. It has also been suggested that the completion of ritualistic stereotypical behaviors may play a role in alleviating anxiety, and that the interruption of stereotypies as may occur in zoological husbandry practices may exacerbate anxiety [28]. The parallels of stereotypical behavior in humans, zoo, laboratory, farmed and companion animals, and the neurological basis of these behaviors in the basal ganglia [28], suggest that such problems in animals may be better considered as mental health disorders rather than simply behavior problems, and should be treated as such.

### 2.4. Addressing Behaviour Problems: Prevention Is Better than Cure

Of primary importance is elucidating and minimizing factors which may trigger ARBs. Table 1 outlines some of the potential triggers which may stimulate ARBs, though this is by no means exhaustive and it is important to acknowledge that the aetiologies of ARBs are not necessarily discrete and may overlap. By considering the potential triggers, and the impact of these triggers on the animal’s cognition, physiology and evolved behaviors, we can see that whilst the symptoms that different animals show may be similar, the potential mechanisms and potential triggers underlying the ARB are varied. Thus, to adequately prevent the development of ARBs we may need to consider a range of factors including but not limited to the animal’s early developmental (and even prenatal) environment [56,57], adequate understanding of and provision for behaviors of evolutionary or neurobiological significance such as those linked to the motivational systems outlined by Panksepp [10,58], and the mitigation of stressors to which the animal is not evolved to cope [10].

As ARBs arise from varied aetiologies, it can often be challenging to elucidate the trigger for the stereotypy, for example, stereotypic digging behavior in gerbils appears to be goal-directed and may be alleviated not by the provision of a sand-digging substrate but by the provision of a tunnel [65]. Thus, the digging behavior may be considered to be a mechanism enabling the gerbil to ‘cope’ better with a lack of a tunnel. Alternatively, it is possible that the lack of environmental stimulation during early cognitive development may also have influenced the performance of stereotypical digging in the young gerbils as the group offered only the sand substrate were not offered it until day 15 of development as opposed to the group offered the more complex tunnel enclosure, given access at day 2 of development [65]. Regardless of the mechanism, it is clear that the burrow/tunnel resource is important to the young of this species and that the development of the digging behavior is related to the absence of this resource. Conversely, in some situations it is the performance of the behavior that seems to be of importance, rather than the acquisition of a specific resource. For example in pursuit predators, such as cheetah and coyotes, the risk of route-tracing and other stereotypical behaviors appears to be linked to their need for large home ranges and long hunting chase distances [10] even when housed in zoo environments where adequate food is assumed to be provided. Animals which are prevented from performing a highly motivated behavior may suffer [66], which is a considerable animal welfare problem.

### 2.5. Environmental Enrichment—A Universal Panacea?

Once an animal is displaying an ARB, we should ideally attempt to elucidate and then mitigate the potential triggers. Often in zoos however, a more disparate approach is employed and this may account somewhat for the lack of long-term success in addressing ARBs in zoo species. The provision of enrichment is a commonly employed mechanism for preventing and treating ARBs. Enrichment may be defined as the provision of choices, designed to stimulate the senses [67], but the value and functionality of specific enrichment items, or their relevance to individual animals may not always be considered when developing enrichment programmes or using enrichment as therapy for ARBs. For example, it is often recommended to offer animals a range of enrichment from human-defined categories, e.g., physical, social, cognitive, food, sensory, etc. [67,68]. However, this approach assumes that these categories make sense to zoo animals and are of equivalent importance to the animals, when in fact this categorization is simply a mechanism to encourage variation in enrichment provision amongst caregivers and may not in fact be meeting the needs of the animal. For example, specific enrichments such as foraging enrichments may be of greater value to maned wolves than novel object enrichments [69].

If we accept that animals have a need to exhibit highly motivated natural behaviors, conserved over many generations and linked to neurobiological reward systems [45], it makes sense that enrichment which provides for these behaviors and has evolutionary relevance may be more successful than simply the provision of variety and novelty. Enrichment can only be considered to be enriching if its impact is evaluated and it is shown to be successful [25,67,70]. Species-appropriate enrichment is preferred [71], and so the functional and evolutionary relevance of enrichment items rather than simply novelty or variety should be considered [70].

In general, environmental enrichment has been suggested to be effective at significantly reducing stereotypy in about half of cases [11], although it is acknowledged that descriptions of stereotypy and enrichment strategies are often poorly defined [11]. A number of frameworks exist for enrichment planning, e.g., the Shape of Enrichment Planning Flow Chart, S.P.I.D.E.R, and are used widely around the world. Whilst such approaches provide a helpful guide to setting goals and developing enrichment strategies, in practice goal setting may deviate from the originally described objectives; improving welfare, successful reproduction, reducing stress, decreasing abnormal and increasing normal behavior, and successful reintroduction [25,71] and diversify into merely providing novelty, particularly in regions where understanding of species-specific ecology may be lacking. Even in well-resourced zoos, zookeepers are not always particularly successful at determining what is likely to be a successful enrichment intervention [70], and this may impact on their ability to provide successful species-appropriate enrichment.

The use of technology in enrichment may be perceived positively by the public but may not always be effective in enhancing the welfare of an animal [72,73]. Technological devices may also create problems in terms of competition [72] or practicality [74]. In some cases, the application of technology in the field of zoo animal enrichment may fail to account for species-specific ecology and thus may fail to address the underlying motivation for the behavioral problem it is aiming to fix. However, technology may also provide useful opportunities to enhance welfare when applied appropriately [75]. As with any enrichment approach, evaluation of success is necessary [25]. Recognizing that enrichment is often applied to prevent or reduce the performance of abnormal repetitive behaviors, it is essential that such enrichment devices address the motivational bases of ARBs rather than simply attempting to ‘distract’ or ‘entertain’ an animal. Despite these reservations, the provisions of environmental enrichment in the absence of identifying specific triggers has some merit [11], and whilst efforts should be focused on identifying and mitigating triggers of ARBs, this does not also mean that effort should not also be made to enhance enrichment provision. The development of technological enrichment devices has the potential to be hugely powerful in providing zoo animals with choice and control and in mitigating behavioral frustration, but it is important that the development of enrichment devices is founded not simply in novelty but in a solid understanding of clinical animal behavior and applied ethology. We must always ask ourselves—is this animal healthy and does it have what it wants? [76], recognizing the basis for ARBs is failure to cope, behavioral frustration, or psychopathology [55], it is essential that behavioral modification to address ARBs addresses the specific motivation for that ARB, rather than applying random novelty which may distract in the short term but is less likely to be successful in the long term. So if enrichment alone is not the answer, what else might we consider?

### 2.6. Behavioural Pharmacology

In companion animals, over 60% of clinical behavioral referrals are linked to pain experiences (pers. comm D. Mills, BSAVA, 2018) and it has been suggested that pain may also be a significantly under-diagnosed issue particularly in older zoo animals [77,78,79] and may result in the development of ARBs [62]. In chronic neuropathic pain cases, environmental enrichment may be useful in mitigating the animal’s pain response [80], although appropriate analgesic therapy should also be used. In all cases of ARB, pain should be considered as a potential trigger, even in relatively young animals—for example zoo-housed bears as young as 15 years old may show signs of painful degenerative joint disease [79,81], and in zoo-housed carnivores with severe pathology, assessment of quality of life prior to euthanasia may be challenging [82], thus the ARBs may potentially act as a useful signal of underlying pain.

**Figure 3:**
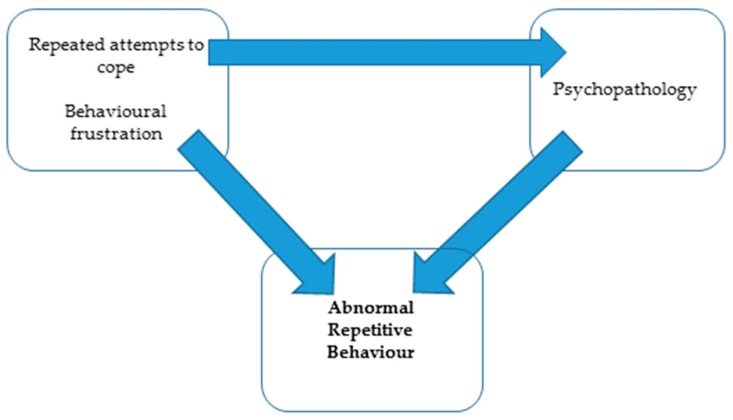
Schematic diagram of the links between repeated attempts to cope, behavioral frustration, psychopathology and Abnormal Repetitive Behaviour.

For ARBs arising from other causes, the use of appropriate pharmacotherapeutic agents alongside appropriate and targeted behavioral modification strategies has been shown to result in better and faster treatment outcomes for problem behaviors in companion animals [83,84]. This approach has also been successful in zoo species including bears [77,85], psitticines [86] and lion (author’s experience). Whilst public and professional concerns exist over the use of psychotherapeutic agents in zoological species, if we accept that some ARBs originate from developmental cognitive pathology, it makes sense that it is necessary to treat this cognitive pathology (psychopathology) in order to adequately manage the ARB. Similarly, if we accept that the ARB may be a response to a stressor or to behavioral frustration and that either of these states may result in physiological stress and the release of cortisol into the circulatory system, we have to consider the physiological consequences of this. ARBs originating from a chronic failure to cope and/or behavioral frustration can lead to cognitive pathology (Figure 3). Specifically, circulating cortisol may act upon the hippocampus in the brain, resulting in temporary amnesia and inhibition of learning or response to new experiences (e.g., enrichment or behavioral modification) [87,88]. Whilst the specific neuroanatomical location that drives motor stereotypies is not yet known, the neurological basis for stereotypies is supported by evidence [55]. Thus, it seems sensible to normalize the brain physiology and chemistry prior to initiating behavioral modification solutions which rely on normal brain functioning. In addition, chronic exposure of the hippocampus to circulating cortisol may accelerate hippocampal degeneration, a normal ageing change associated with senile cognitive dysfunction or dementia-like syndromes which have been described in apes [89], canids [90] and felids [91]. This neurological degeneration may stimulate stereotypy as a symptom of an underlying poor welfare state/behavioral stress/frustration, or may drive of abnormal behaviors as a sign of cognitive dysfunction.

Considering that appropriate behavioral pharmacology in conjunction with appropriate behavioral modification results in better and faster treatment responses, it may be suggested that we have a duty of care to consider pharmacological intervention as one of our primary responses to ARBs rather than a last resort, when other options have failed and where the animal’s welfare may already have been negatively impacted. As discussed above, the management of ARBs should be considered not simply a behavior problem but more specifically as a mental health problem. Once we accept this basis for ARBs, we can understand why enrichment alone may not work as a universal panacea for the management of ARBs and instead focus on more holistic and targeted solutions that have demonstrated greater success in other animal industries.

### 2.7. Framework for Preventing and Managing Behavioural Pathology

**Figure 4:**
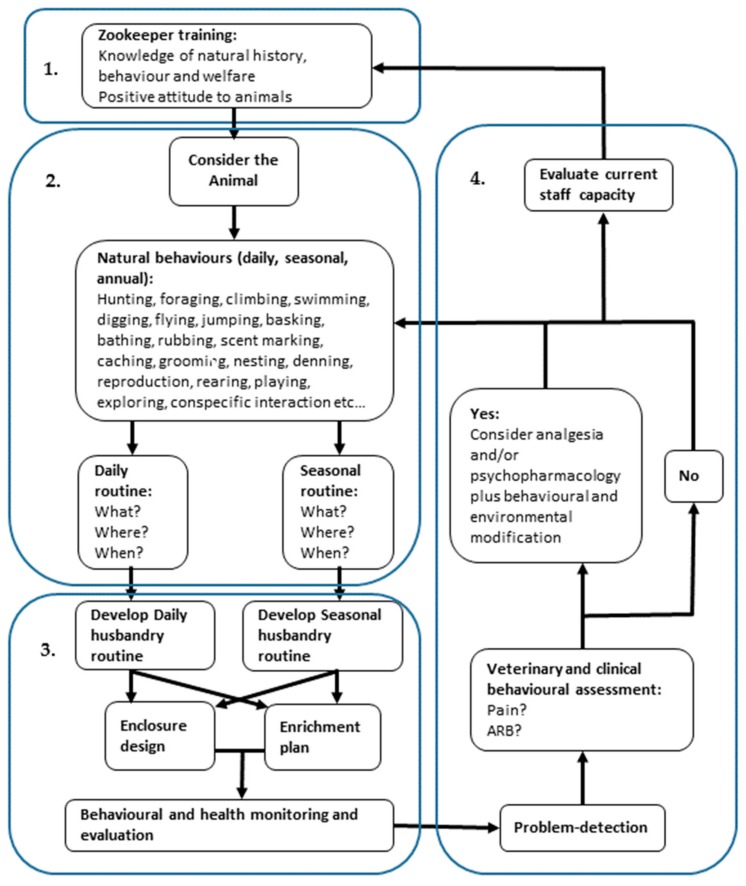
An algorithm for the holistic 4-stage prevention and management of behavioral pathology based on evidence drawn from animal ecology, welfare and clinical animal behavior. (1) Effective and appropriate training of zookeepers to support empathetic attitudes and appropriate species-specific behavioral knowledge. (2) Consideration of the animal, its evolved and species-specific behaviors, its welfare needs and the temporal and spatial provision of resources to it (what is provided, where is provided and when is it provided?). (3) The development of appropriate plans and documentation to facilitate monitoring and evaluation. (4) Detection and management of mental, behavioral or physical health problems.

This paper has presented an array of data from the fields of animal welfare, ecology and clinical animal behavior in order to propose a holistic approach to the prevention and management of behavioral pathologies such as ARBs. This approach is summarized in the algorithm below (Figure 4) and comprises primarily 3 stages: (1) Effective and appropriate training of zookeepers to support empathetic attitudes and appropriate species-specific behavioral knowledge. (2) Consideration of the animal, its evolved and species-specific behaviors, its welfare needs and the temporal and spatial provision of resources to it (what is provided, where is provided and when is it provided?). (3) The development of appropriate plans and documentation to facilitate monitoring and evaluation. An additional multi-step phase (4) is outlined if physical health or behavioral pathologies are detected.

Whilst considerable developments in zoo animal welfare and clinical behavioral medicine have occurred over the recent years, these developments have not always been integrated and may not form part of zookeeper training. Although current proposals to develop a European Zookeeper qualification framework [92] may go some way to mitigating these gaps, there is still a lack of recognition of the clinical significance of behavioral pathology within both zookeepers and zoo veterinarians. Effectively addressing ARBs and supporting good behavioral health in zoo animals must start with comprehensive stockmanship skills and an extensive understanding of species-specific ecology within the zookeeping community. Senior management support is required to ensure that keepers are appropriately trained and resourced and that a culture of care is promoted within the zoo [14], but the practical implementation of effective behavioral management strategies falls primarily to animal-keeping staff, with senior management and veterinary input where required.

## 3. Conclusions

Preventing and managing ARBs is a complex and multifaceted problem requiring understanding of a number of scientific disciplines and integration of veterinary and behavioral management, with resource allocation and staff training. Enhancing animal welfare in many zoos may require moving away from commonly perceived ‘solutions’ and human behavior patterns and towards a more problem-solving elucidation of factors which may be triggering the ARB. The use of a straightforward algorithm in addressing the problem of ARBs allows for a diversity of potentially complex causal factors to be considered whilst providing clear and practical guidance, thus bridging the gap between high-level welfare frameworks and individual enrichment planning tools.

## Figures and Tables

**Table 1:** Suggested triggers and mechanisms for the development of Abnormal Repetitive Behaviours (ARBs) in zoo animals.

Potential Triggers	Suggested Mechanism	Aetiology of ARBs [9,55]	Symptom
Negative human–animal interactions e.g., zoo visitors, zoo staff, chronic pain [59,60,61,62].	The animal does not have the evolved behaviours or physiology to cope with stressors.	Repeated attempts to cope	Abnormal repetitive behaviours, e.g., stereotypy, self-directed behaviours or externally directed behaviours
Frustrated reproductive, maternal, foraging, predatory, browsing, grazing behaviours [10,58,63].	A lack of provisions prevents the animal from performing its evolved behaviours or physiological needs.	Behavioural frustration	
Early maternal separation, barren environment during development [56,57,64].	Impaired cognitive development.	Psychopathology	

## References

[1] Mellor D.J., Hunt S., Gusset M. (2015). Caring for Wildlife: The World Zoo and Aquarium animal Welfare Strategy.

[2] Powell D.M., Watters J.V. (2017). The evolution of the animal welfare movement in US. Zoos and aquariums. Der Zool. Gart..

[3] Kagan R., Veasey J. (2010). Challenges of Zoo Animal Welfare. Wild Mammals in Captivity: Principles and Techniques for Zoo Management.

[4] Carlstead K., Shepherdson D. (1994). Effects of environmental enrichment on reproduction. Zoo Biol..

[5] Tetley C.L., O’Hara S.J. (2012). Ratings of animal personality as a tool for improving the breeding, management and welfare of zoo mammals. Anim. Welf..

[6] Zhang G., Swaisgood R.R., Zhang H. (2004). Evaluation of behavioral factors influencing reproductive success and failure in captive giant pandas. Zoo Biol..

[7] Jule K.R., Leaver L.A., Lea S.E.G. (2008). The effects of captive experience on reintroduction survival in carnivores: A review and analysis. Biol. Conserv..

[8] Reading R.P., Miller B., Shepherdson D. (2013). The value of enrichment to reintroduction success. Zoo Biol..

[9] Rose P.E., Nash S.M., Riley L.M. (2017). To pace or not to pace? A review of what abnormal repetitive behavior tells us about zoo animal management. J. Vet. Behav. Clin. Appl. Res..

[10] Kroshko J., Clubb R., Harper L., Mellor E., Moehrenschlager A., Mason G. (2016). Stereotypic route tracing in captive carnivora is predicted by species-typical home range sizes and hunting styles. Anim. Behav..

[11] Swaisgood R.R., Shepherdson D.J. (2005). Scientific approaches to enrichment and stereotypies in zoo animals: What’s been done and where should we go next?. Zoo Biol..

[12] World Association of Zoos and Aquariums (2005). Building a Future for Wildlife: The World Zoo and Aquarium Conservation Strategy.

[13] Catibog-Sinha C. (2008). Zoo tourism: Biodiversity conservation through tourism. J. Ecotour..

[14] Kagan R., Carter S., Allard S. (2015). A universal animal welfare framework for zoos. J. Appl. Anim. Welf. Sci..

[15] Blackett T.A., McKenna C., Kavanagh L., Morgan D.R. (2017). The welfare of wild animals in zoological institutions: Are we meeting our duty of care?. Int. Zoo Yearb..

[16] Melfi V., Hosey G. (2011). Capacity building for better animal welfare. Int. Zoo Yearb..

[17] Melfi V.A. (2009). There are big gaps in our knowledge, and thus approach, to zoo animal welfare: A case for evidence-based zoo animal management. Zoo Biol..

[18] Goulart V.D., Azevedo P.G., Schepop J.A.V.D., Teixeira C.P., Barçante L., Azevedo C.S., Young R.J. (2009). Gaps in the study of zoo and wild animal welfare. Zoo Biol..

[19] Hill S.P., Broom D.M. (2009). Measuring zoo animal welfare: Theory and practice. Zoo Biol..

[20] Wickins-Dražilová D. (2006). Zoo animal welfare. J. Agric. Environ. Ethics.

[21] Waugh D.R., Wemmer C., Olney P.J.S., Mace G.M., Feistner A.T.C. (1994). Training in zoo biology: Two approaches to enhance the conservation role of zoos in the tropics. Creative Conservation: Interactive Management of Wild and Captive Animals.

[22] BIAZA. https://biaza.org.uk/downloader/78.

[23] Hosey G., Melfi V. (2015). Are we ignoring neutral and negative human–animal relationships in zoos?. Zoo Biol..

[24] Ward S.J., Melfi V. (2015). Keeper-animal interactions: Differences between the behaviour of zoo animals affect stockmanship. PLoS ONE.

[25] Alligood C., Leighty K. (2015). Putting the “e” in spider: Evolving trends in the evaluation of environmental enrichment efficacy in zoological settings. Anim. Behav. Cognit..

[26] Ross S.R. (2006). Issues of choice and control in the behaviour of a pair of captive polar bears (ursus maritimus). Behav. Processes.

[27] Owen M.A., Swaisgood R.R., Czekala N.M., Lindburg D.G. (2005). Enclosure choice and well-being in giant pandas: Is it all about control?. Zoo Biol..

[28] Eilam D., Zor R., Szechtman H., Hermesh H. (2006). Rituals, stereotypy and compulsive behavior in animals and humans. Neurosci. Biobehav. Rev..

[29] Hediger H. (1964). Wild Animals in Captivity.

[30] Réale D., Reader S.M., Sol D., McDougall P.T., Dingemanse N.J. (2007). Integrating animal temperament within ecology and evolution. Biol. Rev..

[31] Sergiel A., Naves J., Kujawski P., Maślak R., Serwa E., Ramos D., Fernández-Gil A., Revilla E., Zwijacz-Kozica T., Zięba F. (2017). Histological, chemical and behavioural evidence of pedal communication in brown bears. Sci. Rep..

[32] Tattoni C., Bragalanti N., Groff C., Rovero F. (2015). Patterns in the use of rub trees by the eurasian brown bear. Hystrix Ital. J. Mammal..

[33] Clapham M., Kitchin J. (2016). Social play in wild brown bears of varying age-sex class. Acta Ethol..

[34] Horner M.A., Powell R.A. (1990). Internal structure of home ranges of black bears and analyses of home-range overlap. J. Mammal..

[35] Penteriani V., López-Bao J.V., Bettega C., Dalerum F., Delgado M.D.M., Jerina K., Kojola I., Krofel M., Ordiz A. (2017). Consequences of brown bear viewing tourism: A review. Biol. Conserv..

[36] Maślak R., Sergiel A., Bowles D., Paśko Ł. (2016). The welfare of bears in zoos: A case study of poland. J. Appl. Anim. Welf. Sci..

[37] Miller S., Wilder J., Wilson R.R. (2015). Polar bear–grizzly bear interactions during the autumn open-water period in alaska. J. Mammal..

[38] Shepherdson D., Lewis K.D., Carlstead K., Bauman J., Perrin N. (2013). Individual and environmental factors associated with stereotypic behavior and fecal glucocorticoid metabolite levels in zoo housed polar bears. Appl. Anim. Behav. Sci..

[39] Heesen R., Genty E., Rossano F., Zuberbühler K., Bangerter A. (2017). Social play as joint action: A framework to study the evolution of shared intentionality as an interactional achievement. Learn. Behav..

[40] Mattiello S., Brignoli S.M., Cordedda A., Pedroni B., Colombo C., Rosi F. (2014). Effect of the change of social environment on the behavior of a captive brown bear (*Ursus arctos*). J. Vet. Behav. Clin. Appl. Res..

[41] Lührs M.-L., Dammhahn M. (2010). An unusual case of cooperative hunting in a solitary carnivore. J. Ethol..

[42] Hull V., Zhang J., Zhou S., Huang J., Li R., Liu D., Xu W., Huang Y., Ouyang Z., Zhang H. (2015). Space use by endangered giant pandas. J. Mammal..

[43] Winter A., Cooper J.J. (2015). Observational Study on the Effects of Enclosure Size and Design on Diurnal Activity and Species-Specific Behaviours in Captive Polar Bear (*Ursus maritimus*).

[44] Woods K., Cooper J.J. (2017). Effects of Seasonal Changes and Climate on the Behaviour of the Polar Bear (*Ursus maritimus*) Population at the Yorkshire Wildlife Park.

[45] Mellor D., Beausoleil N. (2015). Extending the ‘five domains’ model for animal welfare assessment to incorporate positive welfare states. Anim. Welf..

[46] Bashaw M.J., Gibson M.D., Schowe D.M., Kucher A.S. (2016). Does enrichment improve reptile welfare? Leopard geckos (*Eublepharis macularius*) respond to five types of environmental enrichment. Appl. Anim. Behav. Sci..

[47] Cordero-Rivera A. (2017). Behavioral diversity (ethodiversity): A neglected level in the study of biodiversity. Front. Ecol. Evolut..

[48] Kirkwood J.K. (2003). Welfare, husbandry and veterinary care of wild animals in captivity: Changes in attitudes, progress in knowledge and techniques. Int. Zoo Yearb..

[49] Poole T.B. (1988). Normal and abnormal behavior in captive primates. Primate Rep..

[50] Polverino G., Manciocco A., Vitale A., Alleva E. (2015). Stereotypic behaviours in *Melopsittacus undulatus*: Behavioural consequences of social and spatial limitations. Appl. Anim. Behav. Sci..

[51] Schuppli C., Fraser D., Bacon H. (2014). Welfare of non-traditional pets. Rev. Sci. Tech..

[52] Martínez Silvestre A. (2014). How to assess stress in reptiles. J. Exotic Pet Med..

[53] Crane A.L., Ferrari M.C.O. (2017). Learning of safety by a social fish: Applications for studying post-traumatic stress in humans. Anim. Behav..

[54] Greenway E., Jones K.S., Cooke G.M. (2016). Environmental enrichment in captive juvenile thornback rays, *Raja clavata* (linnaeus 1758). Appl. Anim. Behav. Sci..

[55] Mason G.J., Mason G., Rushen J. (2006). Stereotypic behaviour in captive animals: Fundamentals and applications to welfare In Stereotypies in Captive Animals.

[56] Rutherford K.M.D., Piastowska-Ciesielska A., Donald R.D., Robson S.K., Ison S.H., Jarvis S., Brunton P.J., Russell J.A., Lawrence A.B. (2014). Prenatal stress produces anxiety prone female offspring and impaired maternal behaviour in the domestic pig. Physiol. Behav..

[57] Eriksen M.S., Poppe T.T., McCormick M., Damsgård B., Salte R., Braastad B.O., Bakken M. (2015). Simulated maternal pre-spawning stress affects offspring’s attributes in farmed atlantic salmon salmo salar (linnaeus, 1758). Aquac. Res..

[58] Panksepp J. (2011). The basic emotional circuits of mammalian brains: Do animals have affective lives?. Neurosci. Biobehav. Rev..

[59] Fernandez E.J., Tamborski M.A., Pickens S.R., Timberlake W. (2009). Animal and visitor interactions in the modern zoo: Conflicts and interventions. Appl. Anim. Behav. Sci..

[60] Sherwen S.L., Magrath M.J.L., Butler K.L., Hemsworth P.H. (2015). Little penguins, eudyptula minor, show increased avoidance, aggression and vigilance in response to zoo visitors. Appl. Anim. Behav. Sci..

[61] Carlstead K. (2009). A comparative approach to the study of keeper–animal relationships in the zoo. Zoo Biol..

[62] Maslak R., Sergiel A., Hill S.P. (2013). Some aspects of locomotory stereotypies in spectacled bears (tremarctos ornatus) and changes in behavior after relocation and dental treatment. J. Vet. Behav. Clin. Appl. Res..

[63] Mellor D.J. (2015). Positive animal welfare states and encouraging environment-focused and animal-to-animal interactive behaviours. N. Z. Vet. J..

[64] Latham N.R., Mason G.J. (2008). Maternal deprivation and the development of stereotypic behaviour. Appl. Anim. Behav. Sci..

[65] Wiedenmayer C. (1997). Causation of the ontogenetic development of stereotypic digging in gerbils. Anim. Behav..

[66] Dawkins M.S. (1988). Behavioural deprivation: A central problem in animal welfare. Appl. Anim. Behav. Sci..

[67] Hoy J.M., Murray P.J., Tribe A. (2010). Thirty years later: Enrichment practices for captive mammals. Zoo Biol..

[68] European Commission (2015). EU Zoos Directive: Good Practices Document.

[69] Cummings D., Brown J.L., Rodden M.D., Songsasen N. (2007). Behavioral and physiologic responses to environmental enrichment in the maned wolf (chrysocyon brachyurus). Zoo Biol..

[70] Mehrkam L.R., Dorey N.R. (2015). Preference assessments in the zoo: Keeper and staff predictions of enrichment preferences across species. Zoo Biol..

[71] Mellen J., MacPhee M.S. (2001). Philosophy of environmental enrichment: Past, present, and future. Zoo Biol..

[72] Tarou L.R., Kuhar C.W., Adcock D., Bloomsmith M.A., Maple T.L. (2004). Computer-assisted enrichment for zoo-housed orangutans (pongo pygmaeus). Anim. Welf..

[73] Perdue Bonnie M., Clay Andrea W., Gaalema Diann E., Maple Terry L., Stoinski Tara S. (2012). Technology at the zoo: The influence of a touchscreen computer on orangutans and zoo visitors. Zoo Biol..

[74] French F., Mancini C., Sharp H. (2018). High tech cognitive and acoustic enrichment for captive elephants. J. Neurosci. Methods.

[75] Kim-McCormack N.N.E., Smith C.L., Behie A.M. (2016). Is interactive technology a relevant and effective enrichment for captive great apes?. Appl. Anim. Behav. Sci..

[76] Dawkins M.S. (2003). Behaviour as a tool in the assessment of animal welfare1. Zoology.

[77] Bourne D.C., Cracknell J.M., Bacon H.J. (2010). Veterinary issues related to bears (ursidae). Int. Zoo Yearb..

[78] Goldberg M.E. (2017). How to be a pain management advocate for exotic and zoo animals. Vet. Nurse.

[79] Kitchener A., Macdonald A.A. (2002). The longevity legacy: The problem of old animals in zoos. Adv. Ethol..

[80] Vachon P., Millecamps M., Low L., Thompsosn S.J., Pailleux F., Beaudry F., Bushnell C.M., Stone L.S. (2013). Alleviation of chronic neuropathic pain by environmental enrichment in mice well after the establishment of chronic pain. Behav. Brain Funct..

[81] Föllmi J. (2005). Symptoms, Radiographic Examinations and Pathologies: Development of a Scoring System to Evaluate Physical Condition and Quality of Life in Geriatric Zoo Mammals. Uitgever Niet Vastgesteld. http://www.tierschutz.vetsuisse.unibe.ch/e191756/e224004/e224515/e239752/Diss_Foellmi_ger_eng.pdf.

[82] Föllmi J., Steiger A., Walzer C., Robert N., Geissbühler U., Doherr M., Wenker C. (2007). A scoring system to evaluate physical condition and quality of life in geriatric zoo mammals. Anim. Welf..

[83] Overall K.L. (2001). Pharmacological treatment in behavioural medicine: The importance of neurochemistry, molecular biology and mechanistic hypotheses. Vet. J..

[84] Overall K.L. (2005). Proceedings of the dogs trust meeting on advances in veterinary behavioural medicine london; 4th–7th november 2004: Veterinary behavioural medicine: A roadmap for the 21st century. Vet. J..

[85] Yalcin E., Aytug N. (2007). Use of fluoxetine to treat stereotypical pacing behavior in a brown bear (*Ursus arctos*). J. Vet. Behav. Clin. Appl. Res..

[86] Van Zeeland Y. (2018). Medication for behavior modification in birds. Vet. Clin. Exot. Anim. Pract..

[87] Wielebnowski N. (2003). Stress and distress: Evaluating their impact for the well-being of zoo animals. J. Am. Vet. Med. Assoc..

[88] McAuley M.T., Kenny R.A., Kirkwood T.B., Wilkinson D.J., Jones J.J., Miller V.M. (2009). A mathematical model of aging-related and cortisol induced hippocampal dysfunction. BMC Neurosci..

[89] Lowenstine L.J., McManamon R., Terio K.A. (2015). Comparative pathology of aging great apes: Bonobos, chimpanzees, gorillas, and orangutans. Vet. Pathol..

[90] Schütt T., Helboe L., Pedersen L.Ø., Waldemar G., Berendt M., Pedersen J.T. (2016). Dogs with cognitive dysfunction as a spontaneous model for early alzheimer’s disease: A translational study of neuropathological and inflammatory markers. J. Alzheimer's Dis..

[91] Head E., Gunn-Moore D., Landsberg G., Maďari A., Žilka N. (2017). Neuropathology of feline dementia. Canine and Feline Dementia: Molecular Basis, Diagnostics and Therapy.

[92] European Professional Zookeeper Qualification Framework. https://www.zookeepers.eu/framework/area-2-animal-management/2-2-animal-behaviour/#2.2.4.

